# Adenosine Monophosphate-Activated Protein Kinase Signaling Regulates Lipid Metabolism in Response to Salinity Stress in the Red-Eared Slider Turtle *Trachemys scripta elegans*

**DOI:** 10.3389/fphys.2019.00962

**Published:** 2019-07-31

**Authors:** Meiling Hong, Na Li, Jiangyue Li, Weihao Li, Lingyue Liang, Qian Li, Runqi Wang, Haitao Shi, Kenneth B. Storey, Li Ding

**Affiliations:** ^1^Ministry of Education Key Laboratory for Ecology of Tropical Islands, College of Life Sciences, Hainan Normal University, Haikou, China; ^2^Chengdu Institute of Biology, Chinese Academy of Sciences, Chengdu, China; ^3^Department of Biology, Carleton University, Ottawa, ON, Canada

**Keywords:** *Trachemys scripta elegans*, salinity adaptation, fatty acid oxidation, lipid synthesis, lipid metabolism-related transcription factors

## Abstract

Aquatic animals have developed various mechanisms to live in either hyperionic or hypoionic environments, and, as such, not many species are capable of surviving in both. The red-eared slider turtle, *Trachemys scripta elegans*, a well-known freshwater species, has recently been found to invade and inhabit brackish water. Herein, we focus on some of the metabolic adaptations that are required to survive and cope with salinity stress. The regulation of the adenosine monophosphate (AMP)-activated protein kinase (AMPK), a main cellular “energy sensor,” and its influence on lipid metabolism were evaluated with a comparison of three groups of turtles: controls in freshwater, and turtles held in water of either 5‰ salinity (S5) or 15‰ salinity (S15) with sampling at 6, 24, and 48 h and 30 days of exposure. When subjected to elevated salinities of 5 or 15‰, *AMPK* mRNA levels and AMPK enzyme activity increased strongly. In addition, the high expression of the peroxisome proliferator activated receptor-α (*PPARα*) transcription factor that, in turn, facilitated upregulation of target genes including carnitine palmitoyltransferase (*CPT*) and acyl-CoA oxidase (*ACO*). Furthermore, the expression of transcription factors involved in lipid synthesis such as the carbohydrate-responsive element-binding protein (*ChREBP*) and sterol regulatory element-binding protein 1c (*SREBP-1c*) was inhibited, and two of their target genes, acetyl-CoA carboxylase (*ACC*) and fatty acid synthase (*FAS*), were significantly decreased. Moreover, exposure to saline environments also increased plasma triglyceride (TG) content. Interestingly, the content of low-density lipoprotein cholesterol (LDL-C) and total cholesterol (TC) in plasma was markedly higher than the control in the S15 group after 30 days, which indicated that lipid metabolism was disrupted by chronic exposure to high salinity. These findings demonstrate that activation of AMPK might regulate lipid metabolism in response to salinity stress through the inhibition of lipid synthesis and promotion of lipid oxidation in the liver of *T. s. elegans*. This may be an important component of the observed salinity tolerance of these turtles that allow for invasion of brackish waters.

## Introduction

Salinity is a crucial environmental stress factor for aquatic species and can disrupt electrolyte balance, cell energetics, and various other physiological responses (Verity and Smetacek, 1996). Shifts from optimal salinity cause stress in aquatic organisms and increase the energy demand for osmoregulation (Devreker et al., 2009). Lipid metabolism is highly dependent on the cellular energy (adenosine triphosphate – ATP), and the *de novo* lipogenesis pathway requires cytosolic citrate from mitochondria in positive nutrition status (Osellame et al., 2012). Changes in lipid content and fatty acid composition were associated with different salinities (0, 7, 14, 21, and 28 ppt) in juvenile American shad (*Alosa sapidissima*; Liu et al., 2016). However, upon environmental stressors, the normal function of mitochondria can be disrupted, leading to lipid metabolism dysfunction in aquatic animals. Thus, it is important to understand lipid metabolism in-depth to further understand the mechanisms behind biological processes involving the lipid metabolic pathway.

The adenosine monophosphate (AMP)-activated protein kinase (AMPK) is regarded as the energy sensor of cells and plays a very important role in regulating fuel metabolism for anabolic versus catabolic purposes (Ewart and Kennedy, 2011; Hardie et al., 2012). The regulation of energy metabolism *via* AMPK-mediated signaling is an active area of research that ranges from studies of the protein kinase itself (structure, cellular distribution, activity) to its roles in multiple organisms including mammals, turtles, frogs, fish, shrimps, and crabs (Mihaylova and Shaw, 2011; Hardie, 2015; Xu et al., 2016). These studies, among others, have emphasized its role in maintaining energy equilibrium, particularly in regulating hepatic lipid metabolism (Towler and Hardie, 2007; Mihaylova and Shaw, 2011). Interestingly, studies with animal models that show natural metabolic rate depression have found that AMPK signaling is involved in the anoxia tolerance of *T. s. elegans* (Rider et al., 2009) and in freezing and dehydration tolerance of wood frogs (*Rana sylvatica*; Horman et al., 2005; Rider et al., 2006). AMPK phosphorylation can inhibit enzymes related to fatty acid, glycogen, protein, and cholesterol synthesis while stimulating enzymes of glycolysis and lipolysis (Hardie and Pan, 2002; Horman et al., 2002; Munday, 2002). In addition to post-translational regulation of various rate-limiting enzymes, AMPK also regulates the gene expression of key glycolytic and lipogenic enzymes *via* regulating the carbohydrate-responsive element-binding protein (*ChREBP*) and sterol regulatory element-binding protein 1c (*SREBP-1c*) transcription factors, respectively (Kawaguchi et al., 2002; Kohjima et al., 2008), to allow the incorporation of fatty acids into triglycerides that function as a long-term energy reservoir (Dentin et al., 2005).

In recent years, the freshwater turtle, red-eared slider (*Trachemys scripta elegans*), has successfully invaded five continents, and this has been in part due to its strong ability to adapt to diverse environments (Semenov, 2010). Field surveys have indicated that *T. s. elegans* can inhabit and lay eggs in the estuary of the Nandujiang river (Hainan Province, China) and that their hatchlings can be found at the shore of brackish water (Yang and Shi, 2014). Variations of sea surface salinity and temperature were negatively correlated during the deglaciation induced by climate warming, which is expected to reduce surface salinity and expand the brackish water regions (Paterne et al., 1993; Groisman et al., 1999). Therefore, the potential invasion danger of *T. s. elegans* would presumably be serious.

Our previous studies have shown that *T. s. elegans* can survive for more than 3 months in saline environments (<15‰; Hong et al., 2014) or promote the antioxidant defense system against the oxidative stress induced by salinity (Ding et al., 2019) and that during this time, they increase their plasma triglyceride and glucose levels, probably supporting increased fuel/energy needs for survival in the saline environment (Shu et al., 2012). Transcriptomic analyses of *T. s. elegans* exposed to saline environments have shown that lipid metabolism pathways are differentially regulated, relative to controls in freshwater, suggesting a strong link between lipid metabolism and salinity-tolerance (Hong et al., 2018). However, the molecular mechanisms behind this targeting of lipid metabolism in *T. s. elegans* under salinity stress remain unknown. The objective of this study was to investigate the role of AMPK in the regulation of lipid metabolism in *T. s. elegans* under salinity stress. Understanding the molecular mechanisms of lipid metabolism in *T. s. elegans* under salinity-responsive *AMPK* signaling can provide a basis for studying the molecular mechanism of salinity tolerance.

## Materials and Methods

### Experimental Animals and Salinity Exposure

*T. s. elegans* (3 years old, female, mean mass 392.26 ± 20.57 g) was purchased from the Hongwang turtle farm (Haikou, China). Turtles were acclimated in a cement pool (190 cm × 65 cm × 32 cm) at room temperature with dechlorinated water for 2 weeks prior to salinity exposure. Two treatment groups with salinities of 5‰ (S5) and 15‰ (S15) were generated using sea salt crystals and measured with a digital high precision electronic salt meter (Taihua, Chengdu, China). An equal volume of dechlorinated freshwater was added to another tank for the control group. Ceramic tiles were placed in each pond for shelter and basking. Turtles were fed with a commercial diet twice a week, and any remaining food was removed and the water changed in the pools after 24 h. The salinity of the experimental groups was monitored and adjusted accordingly each day. Water temperature in pools was maintained near 26°C, and 12–15 h of light was received by these turtles daily.

Six turtles from each pool were randomly sampled at indicated time of experimental exposure (6, 24, and 48 h and 30 days) and were anesthetized by freezing for 20 min. Blood was sampled from cardiovascular vessels in heart. Heart, liver, skeletal muscle, kidney, lung, and intestine were quickly sampled, frozen in liquid nitrogen, and stored at −80°C until further use. All experimental procedures had the prior approval of the Hainan Provincial Ecological Environment Education Center Experimental Animal Ethics Committee and were conducted under standard protocols for the care and use of laboratory animals at Hainan Normal University (No. HNECEE-2014-004).

### RNA Extraction and Quantitative Real-Time Polymerase Chain Reaction

Total RNA was extracted from heart, liver, skeletal muscle, kidney, lung, and intestine using Trizol (Invitrogen, Carlsbad, USA), according to the manufacturer’s protocol. The quality and concentration of total RNA were assessed using a NanoDrop™ One/OneC spectrophotometer (Thermo Fisher, MA, USA), and the integrity of the RNA was determined by electrophoresis on a 1.2% agarose gel that showed the presence of two sharp 18S and 28S rRNA bands. Approximately 1 μg of total RNA was reverse transcribed to cDNA in 20 μl reactions using Prime Script Reverse Transcriptase (TaKaRa, Tokyo, Japan) according to the manufacturer’s instructions.

Primers for *T. s. elegans* mRNA were designed using Primer-BLAST (Table 1). Relative mRNA expression levels were measured using quantitative real-time polymerase chain reaction (qRT-PCR) analysis using the TB Green QuantiTect RT-PCR Kit (TaKaRa, Tokyo, Japan) and a LightCycler®480 Real-Time PCR System (Roche Diagnostics, Basel, Switzerland). The melting curve, amplification curve, and standard curve of each gene were gained by Roche Light Cycler® 480II Real-Time PCR software. The correlation coefficient of standard curves was >0.99, and all genes amplification efficiency were 98–99%. The following transcripts were examined: acetyl-CoA carboxylase (*ACC*), fatty acid synthase (*FAS*), stearoyl CoA desaturase (*SCD*), carnitine palmitoyltransferase 1 (*CPT-1*), carnitine palmitoyltransferase 2 (*CPT-2*), acyl-CoA oxidase (*ACO*), and acyl-CoA synthetase long-chain 1 (*ACSL-1*). These were analyzed by the 2^−△△Ct^ method of processing data after normalization to *β-actin* as the reference gene (*β-actin* was expressed in the three groups and unaffected under salinity stress). The values of control group at 6 h were standardized to 1.0.

**Table 1:** The specific primer for *T. s. elegans*.

Genes	Accession number in GenBank	Primer sequence (5′–3′)	Product length (bp)
*β-actin*	FJ514826.1	F: GCACCCTGTGCTGCTTACA R: CACAGTGTGGGTGACACCAT	190
*AMPKα1*	XM_005282144.3	F: TGGAGCAGTGGGGTTATTCTC R: CCCGAATGTCTCTGATTGTAGC	199
*AMPKα2*	XM_005284772.3	F: GCTGATTTTGGGTTGTCGAA R: TTGAGTGGGTCAACCTGCA	299
*AMPK-β1*	XM_005288707.2	F: GCTTACTGGGAGGAGAGCGA R: AGGCACTCATCCAGCTCCTG	171
*AMPK-β2*	XM_005311511.3	F: GGCTCGTCCCACTGTCATAC R: CTCGGGCAGGTCCAGAATAG	136
*AMPK-γ1*	XM_008173877.2	F: GCTGACCATCACCGACTTCA R: CCAGCGGTTTGAAGGAGTCT	134
*AMPK-γ2*	XM_024114117.1	F: TCTCCAGCACCGTTCTCAGT R: TCAGAGAGGGAGATGATACCCA	156
*AMPK-γ3*	XM_008167850.2	F: TGGCCCTGGAGATCTTCGT R: TAGGTCTTCTGGGCAGCTAGG	121
*ChREBP*	XM_005294339.2	F: GTCTGTCCCCATCACACTGG R: GGAGTGATGCAGCGAGATCC	166
*SREBP-1c*	XM_008164848.2	F: CCAGTGCGAGAAAGCCAG R: CTCAGCTGTTGGCTGACAT	162
*PPARα*	XM_024100408.1	F: TGCCAAATCTATCCCTGGCTTC R: AGGCTACCAGCATCCCATCT	131
*ACC*	XM_005298822.3	F: GCCAGCTGAAGGACAACACT R: GCATGGTGGAGTGAACGAGT	185
*FAS*	XM_005283095.3	F: CGTTGGATCAGCACCTCCAT R: GCAATCTCCACCACAACAGC	155
*SCD-1*	XM_005285549.3	F: CTGTTCCCAACCCTAGTGCC R: GGCTCCAAATGTGACGAAGC	195
*CPT-1*	XM_005312860.2	F: TTATCCATGCCACCCTGCTC R: CTCCTGGGATACGGGAGGTA	135
*CPT-2*	XM_005284832.3	F: GCTGTCACCCCACAATCTCA R: TCGAGAGTCAAGGTTTCCACAG	146
*ACO*	XM_005297505.3	F: GTTGCAGTGGCCTTCCTGAT R: TGTTGCCCTGAGAGGTCGTT	178
*ACSL-1*	XM_005281979.2	F: TCTGACAAGGCCAAGCTGC R: CTTCTGGATTTGGAGGCACAGG	199
*L-FABP*	XM_008169713.1	F: TGATCCAAAAGGGCAAGGACA R: CTCTCCAGTTGGGCTCTCCA	149

### Biochemical Analysis

Liver tissue (200 mg) was homogenized in 2 ml 0.7% saline and centrifuged at 3,000 × *g* for 15 min at 4°C. The protein-containing supernatant was used to measure enzyme activities (AMPK, ACC, FAS, CPT-1, and ACO) using customized ELISA kits (Zike Biological Technology Co., Ltd., Shenzhen, China) according to the manufacturer’s instructions.

The concentration of plasma triglycerides (TG), high-density lipoprotein cholesterol (HDL-C), low-density lipoprotein cholesterol (LDL-C), and total cholesterol (TC) was measured within 24 h of blood collection in a refrigerator by an Olympus AU640 automatic biochemical analyzer (Olympus, Tokyo, Japan).

### Data Analysis

All experimental data are expressed as mean ± SE and were analyzed by Excel 2016 and SPSS 16.0 statistical software. The interaction of two factors (salinity and time) was verified by two-way analysis of variance. After analyzed homoscedasticity of variances, one-way analysis of variance (ANOVA) was run to examine the influence of environmental salinity. Significance differences between different groups were assessed using LSD multi-comparison, and a *p* lower than 0.05 was considered statistically significant.

## Results

### Tissue-Specific mRNA Expression of *AMPK* Subunits

**Figure 1:**
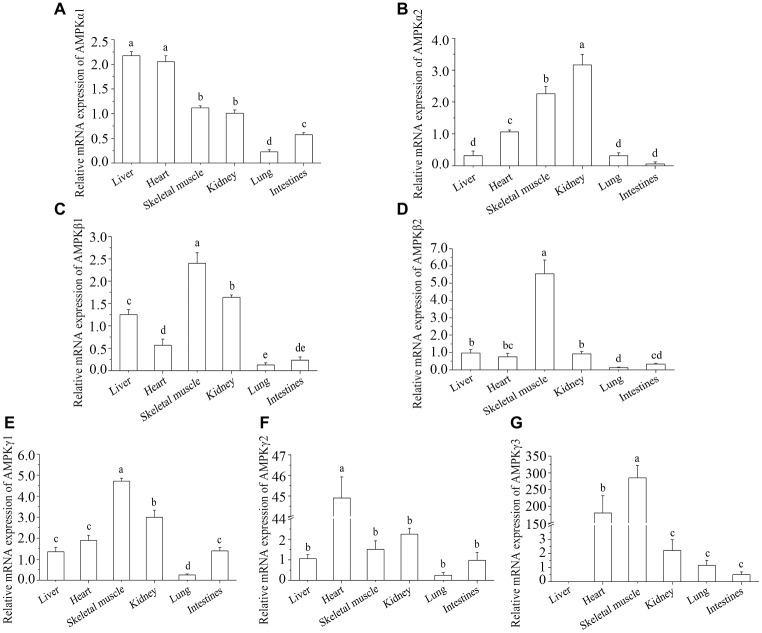
Tissue distribution of *AMPK* subunits in control turtles, *T. s. elegans*. **(A)** *AMPKα1*; **(B)** *AMPKα2*; **(C)** *AMPKβ1*; **(D)** *AMPKβ2*; **(E)** *AMPKγ1*; **(F)** *AMPKγ2*; **(G)** *AMPKγ3*. Note: The ordinate shows the fold change in the relative mRNA expression of AMPK subunits in tissues relative to liver. The superscripts a, b, and c indicate significant differences among tissues.

*AMPK* is a heterotrimeric complex with one catalytic (*α*) and two regulatory (*β* and *γ*) subunits. The tissue-specific expression of *AMPK* subunit mRNA in control turtles is presented in Figure 1. The expression of *AMPKα1* mRNA was highest in liver and heart and the lowest in lung (Figure 1A). The levels of *AMPKα2* mRNA were highest in kidney, with skeletal muscle and heart being the second most abundant, and the other three tissues showing the lowest levels of this subunit (Figure 1B). The mRNA expression levels of *AMPKβ1* were from highest to lowest: skeletal muscle, kidney, liver, heart, intestine, and lung (Figure 1C). The mRNA expression levels of *AMPKβ2* were highest in skeletal muscle and lowest in intestine and lung (Figure 1D). The mRNA expression levels of *AMPKγ1* and *AMPKγ3* were highest in skeletal muscle (Figures 1E,G), and *AMPKγ2* was highest in heart (Figure 1F). However, there was no expression in liver for *AMPKγ3* (Figure 1G).

### *AMPKα* Gene Expression and Adenosine Monophosphate-Activated Protein Kinase Activity Changes After Salinity Stress

**Figure 2:**
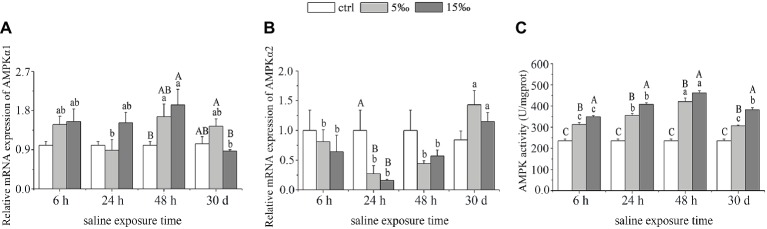
AMPK response to salinity stress in liver. **(A)** mRNA expression of *AMPKα1*; **(B)** mRNA expression of *AMPKα2*; and **(C)** activity of AMPK. Different uppercase letters (A, B, and C) represent significant differences between different salinity groups at the same time point, and different lowercase letters (a, b, and c) represent significant differences between different stress times at the same salinity, *p* < 0.05.

The mRNA expression level of *AMPKα1* in liver of *T. s. elegans* increased markedly only in S15 group at 48 h when compared with the control (*p* = 0.028), whereas S5 group showed no statistical differences at each sampling time. With exposure time extended, there was a peak at 48 h, and significant differences were found in S5 group between 48 and 24 h (*p* = 0.027) and in S15 group between 48 h and 30 days (*p* = 0.009; Figure 2A). Compared with the control, the expression level of *AMPKα2* decreased markedly in S5 and S15 groups at 24 h (*p =* 0.031, *p =* 0.017), began rising again at 48 h (*p =* 0.082, *p =* 0.172), and returned to baseline expression levels at 30 days (*p =* 0.051, *p =* 0.270; Figure 2B). As for AMPK activity, it increased with salinity increased. With exposure time extended, there was a peak at 48 h (Figure 2C). Moreover, there was extremely remarkable interaction effect between exposure time and salinity as for AMPK activity (*F* = 10.636, *p =* 0.000).

### Relative Expression of *AMPK*-Responsive Lipid Metabolism Transcription Factors in Liver

**Figure 3:**
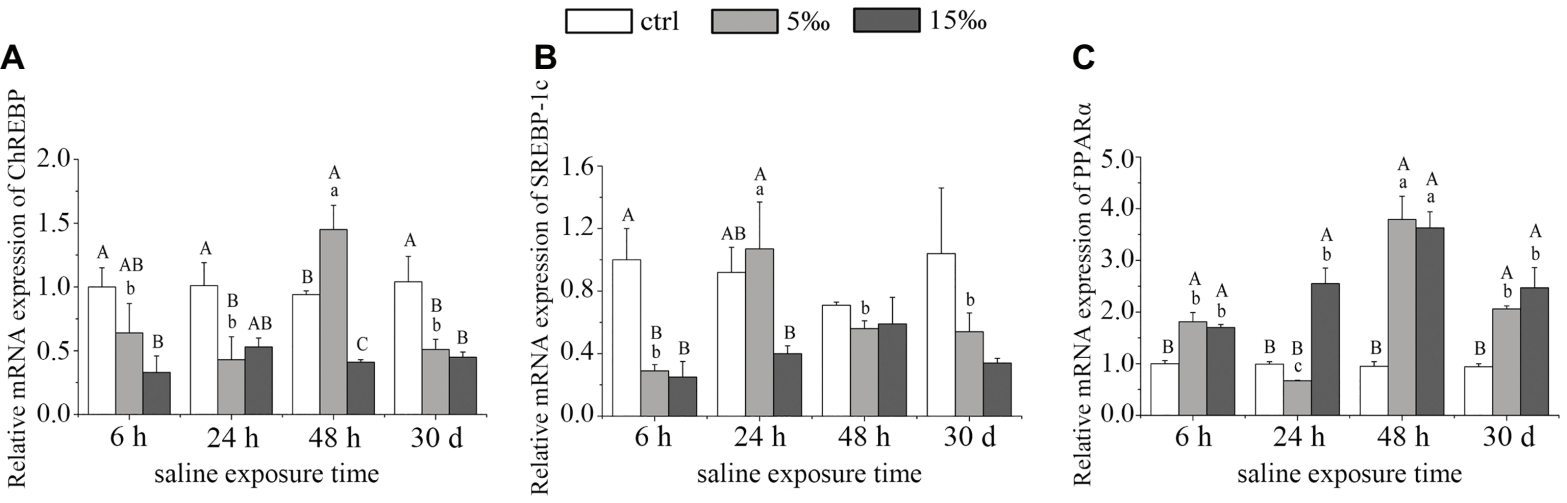
AMPK activates the expression of major lipid metabolism transcription factors in liver of salinity-stressed turtles. **(A)** mRNA expression of *ChREBP*; **(B)** mRNA expression of *SREBP-1c*; and **(C)** mRNA expression of *PPARα*. Different uppercase letters (A, B, and C) represent significant differences between different salinity groups at the same time points, and different lowercase letters (a, b, and c) represent significant differences between different stress times at the same salinity, *p* < 0.05.

As shown in Figure 3, *ChREBP* expression decreased in the liver of S5 group after 24 h (*p* = 0.025) but then rose to maximum expression at 48 h (*p* = 0.012) before decreasing by 30 days (*p* = 0.014), relative to the control. In S15 group, *ChREBP* expression showed a marked decrease except at 24 h (*p* = 0.051; Figure 3A). As for *SREBP-1c* expression in liver, there were significant differences only between S5 and the control at 6 h (*p* = 0.004) and between S15 and the control at 6 h (*p* = 0.003; Figure 3B). By contrast, the mRNA expression level of *PPARα* exhibited a significant increasing over time except S5 group at 24 h, with 48 h exposures displaying the highest abundance (Figure 3C). The mRNA expression levels of *ChREBP* and *PPARα* were highly significant in the interaction effects of exposure time and salinity (*F* = 4.178, *p =* 0.003; *F* = 10.955, *p =* 0.000).

### Relative Expression of Lipid Metabolism Genes in Liver

**Figure 4:**
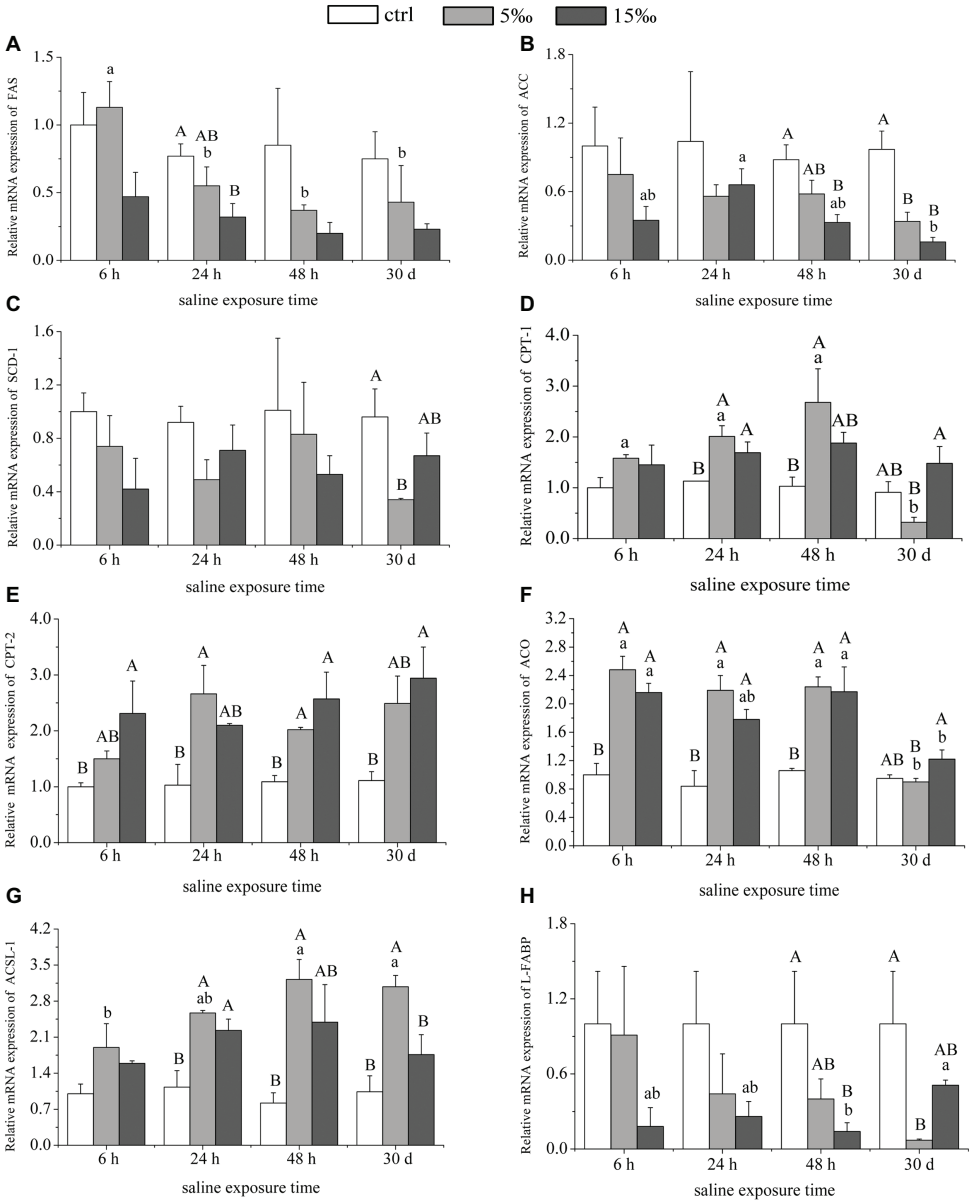
The expressions of lipid metabolism genes in liver. **(A–C)** mRNA expression levels of genes involved in lipid synthesis: *FAS*, *ACC*, and *SCD-1*. **(D–H)** mRNA expression levels of genes involved in lipid oxidation: *CPT-1*, *CPT-2*, *ACO*, *ACSL-1*, and *L-FABP*. Different uppercase letters (A, B, and C) represent significant differences between different salinity groups at the same time point, and different lowercase letters (a, b, and c) represent significant differences between different stress times at the same salinity, *p* < 0.05.

In S15 group, the mRNA expression level of *ACC* in liver decreased 3–4-fold after 48 h (*p* = 0.0006) and 30 days (*p* = 0.000) compared with the control, and *FAS* decreased at 24 h (*p* = 0.020; Figure 4). In S5 group, there was a significant decrease in *ACC* mRNA expression at 30 days (*p* = 0.002), but no significant differences were found in *FAS* mRNA expression, compared with the control (Figures 4A,B). Relative mRNA abundance of stearoyl CoA desaturase 1 (*SCD1*) decreased in S5 group only after 30 days (*p* = 0.021), and there was no significant difference between the other groups (Figure 4C). The mRNA expression levels of *CPT-1*, *CPT-2*, *ACO*, and *ACSL-1* showed a significant trend of enhanced abundance with increased salinity exposures, relative to the control (Figures 4D–G). However, the expression level of *L-FABP* decreased by almost 5-fold in S5 group as the exposure time increased to 30 days (*p* = 0.019; Figure 4H). Only *CPT-1* and *ACO* among them were controlled by the interaction effects of salinity and exposure time dramatically (*F* = 3.323, *p =* 0.010; *F* = 4.346, *p =* 0.002).

### The Enzymatic Activities of Lipid Metabolism-Related Enzymes in Liver

**Figure 5:**
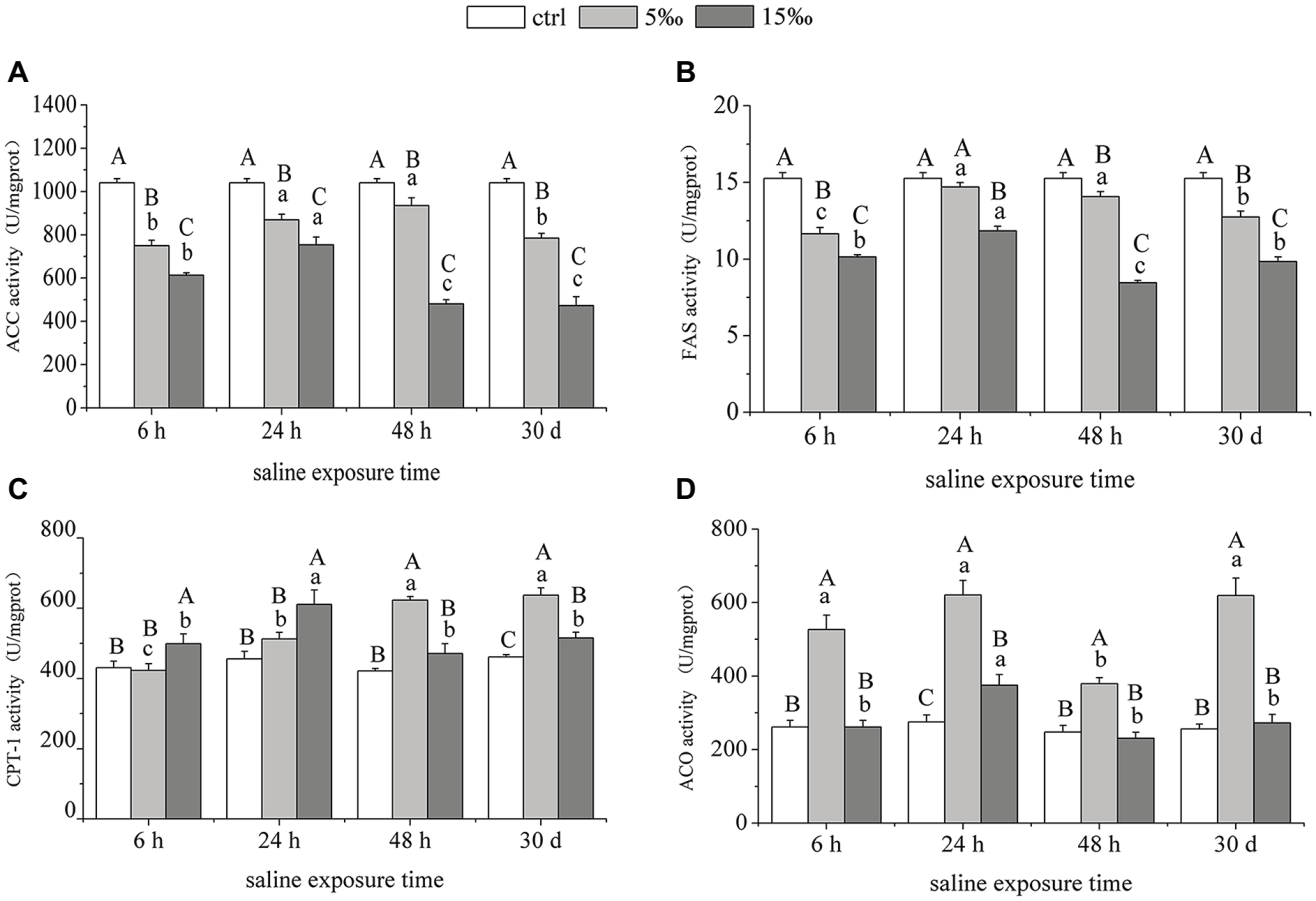
Enzymatic activities of lipid metabolism-related enzymes in liver under salinity stress. **(A,B)** ACC and FAS. **(C,D)** CPT-1 and ACO. Different uppercase letters (A, B, and C) represent significant differences between different salinity groups at the same time point, and different lowercase letters (a, b, and c) represent significant differences between different stress times at the same salinity, *p* < 0.05.

The activities of ACC and FAS in liver decreased strongly as ambient salinity increased (Figures 5A,B). Activity of CPT-1 related to lipid oxidation increased significantly in S5 group at 48 h (*p* = 0.000) and 30 days (*p* = 0.000) and also increased in S15 group at 6 h (*p* = 0.045), 24 h (*p* = 0.002), and 30 days (*p* = 0.028), relative to control levels (Figure 5C). However, ACO activity in S5 was higher than that in S15 and the control, and there was no significant difference between S15 and the control, except after 24 h salinity treatment (Figure 5D). Analysis of variance results shows that there was a significant interaction effect within salinity and exposure time on the activities of ACC, FAS, CPT-1, and ACO (*F* = 12.388, *p* = 0.000; *F* = 10.666, *p* = 0.000; *F* = 10.350, *p* = 0.000; *F* = 4.640, *p* = 0.001).

### Lipid Markers in Plasma: Triglyceride, Low-Density Lipoprotein Cholesterol, High-Density Lipoprotein Cholesterol, and Total Cholesterol

**Figure 6:**
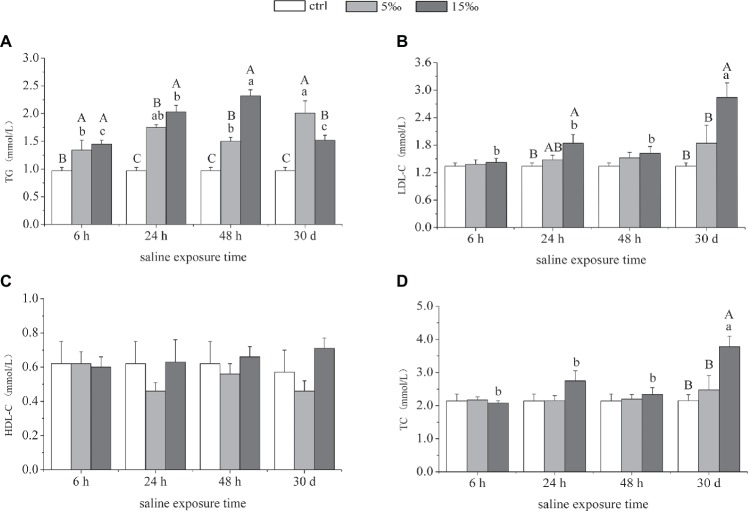
Lipid contents in plasma in response to salinity exposure in *T. s. elegans*. Levels of TG **(A)**, LDL-C **(B)**, HDL-C **(C)**, and TC **(D)**. Different uppercase letters (A, B, and C) represent significant difference between different salinity groups at the same time, and different lowercase letters (a, b, and c) represent significant difference between different stress time at the same salinity, *p* < 0.05.

The content of TG in plasma increased markedly as salinity increased, and the levels in both salinity treatment groups were highest at 24 h (*p* = 0.000, *p* = 0.000) and 48 h (*p* = 0.001, *p* = 0.000), relative to the control (Figure 6A). The contents of plasma LDL-C and TC in S15 group were notably higher than the other two groups after 30 days (Figures 6B,D). There was no significant difference in the content of HDL-C among these three groups over the course of the different salinity exposures (Figure 6C). As for the interaction effects between salinity and exposure time, there were significant effects on the contents of plasma TG, LDL, and TC (*F* = 7.719, *p* = 0.000; *F* = 3.412, *p =* 0.007; *F* = 2.981, *p =* 0.015).

## Discussion

### The mRNA Expression and Activity of Adenosine Monophosphate-Activated Protein Kinase During Salinity Stress

An analysis of tissue-specific gene expression is useful to achieve a better understanding of physiological role of a gene and its protein product. *AMPK* is a heterotrimeric complex composed of the catalytic *α*, the scaffolding *β*, and the nucleotide binding *γ* subunits that are widely distributed in all tissues (Steinberg and Kemp, 2009). The enzyme plays an important role in both regulating intracellular energy balance and the cellular response to stress (Park et al., 2002). There are two or three isoforms of each subunit in mammalian tissues (*α1* and *α2*; *β1* and *β2*; *γ1*, *γ2,* and *γ3*), each encoded by a different gene. In our study, mRNA transcripts of all seven subunits were detected in *T. s. elegans* tissues. The expression of *AMPKα1* was highest in liver, and this corresponds with analogous findings in fish and mammals, where *AMPKα1* was highly expressed in metabolically active tissues, such as liver, kidney, and brain (Stapleton et al., 2016). In this study, *AMPKα2* expression was higher in kidney, skeletal muscle, and heart. Previous research reported that *AMPKα2* plays an important role in glucose metabolism in mammalian muscles (Jørgensen et al., 2007); however, elucidating the regulatory role of *AMPKα2* on glucose metabolism in turtles still requires further study. The *β1* and *β2* subunits were also higher in skeletal muscle of *T. s. elegans* than other tissues. They provide the scaffold on which the *α* and *γ* subunits assemble (Hardie et al., 1998). Of the *γ* isoforms, *γ1* and *γ2* were expressed quite uniformly across all tissues examined, with the exception of low level of *γ1* in lung and high level of *γ2* in heart. As for the mRNA expression level of *γ3*, it was mainly detected in skeletal muscle and heart of the tissues examined; however, there was no expression in liver. The result is in accord with the study in mammalian in which significant expression of *γ3* mRNA was found only in skeletal muscle (Cheung et al., 2000). Previous work suggested that there were no selective associations among the *α1* and *α2* subunits; the *β1* and *β2* subunits; and the *γ1*, *γ2,* or *γ3* subunits (Thornton et al., 1998). Therefore, the catalytic subunits, *AMPKα1* and *AMPKα2*, in liver were selected for further analysis to attempt to determine if they had separate functions in the regulation of lipid metabolism in *T. s. elegans* under salinity stress.

Salinity exposures were found to significantly alter the gene expression levels of *AMPKα1* and *α2* subunits in a salinity- and time-dependent manner, suggesting that *AMPKα1* and *α2* are involved in mediating metabolic responses to salinity stress (Zeng et al., 2016). Indeed, studies on other salt-stressed organisms, such as the Pacific white shrimp *Litopenaeus vannamei*, found that *AMPKα* mRNA levels were upregulated in response to elevated nitrite levels in water (Xu et al., 2016). Interestingly, in the present study, the activity of *AMPK* was significantly increased by salinity stress and was found to closely resemble the mRNA expression level of *AMPKα1*. Future studies should explore the role of phosphorylation in *AMPK* activation during salinity stress of turtle by examining the reversible phosphorylation at Thr172 within the activation loop of the α-subunit, as this posttranslational modification is the most potent activator of AMPK (Oakhill et al., 2012).

### Lipid Metabolism Activated by *AMPK* Signaling During Salinity Stress

The activation of *AMPK* can directly phosphorylate *SREBP-1c* and *ChREBP*, both of which are important lipid-related transcription factors that enter the nucleus and activate the transcription of lipogenic genes in liver (Yamashita et al., 2001). In recent years, *ChREBP* has emerged as an important regulator of glycolytic and lipogenic genes by binding to glucose-responsive DNA elements, termed carbohydrate response elements (*ChRE*), in the promoter regions of selected genes (Rufo et al., 2001). In our study, the gene expression of *SREBP-1c* and *ChREBP* shows a general decreasing trend as salinity increased, indicating the potential suppression of the downstream lipogenic genes under its control. Interestingly, the reduced level of *ChREBP* during salinity stress was different from the response turtles during anoxia. Indeed, when exposed to 5 h of acute anoxia, both transcript and protein levels of *ChREBP* increased in liver and kidney of *T. s. elegans* (Krivoruchko and Storey, 2014).

*SREBP-1c* is also able to induce lipogenic genes through its ability to bind to sterol regulatory elements (*SREs*) present in gene promoters (Koo et al., 2001). Transgenic mice that over-express *SREBP-1c* in liver exhibited liver steatosis and increased mRNA of most lipogenic genes (Shimano et al., 1997). With this in mind, the decrease in *SREBP-1c* transcript level with increased salinity suggests that the lipogenic enzyme that it controls would also be reduced by salinity stress. *SREBP-1c*, together with *ChREBP*, preferentially regulates the lipogenic process by activating genes involved in fatty acid and triglyceride synthesis such as acetyl-CoA carboxylase (*ACC*), fatty acid synthase (*FAS*), and stearoyl CoA desaturase (*SCD*; Dentin et al., 2005; Xu et al., 2013). In our study, the transcript levels of *ACC*, *FAS,* and *SCD-1* were significantly decreased, along with their enzyme activities, suggesting that salinity stress is playing a role to reduce lipogenesis in the liver of *T. s. elegans*. *ACC* is a well-defined substrate of *AMPK*, converts acetyl-CoA to malonyl-CoA, and is highly expressed in lipogenic tissues such as liver and adipose where it regulates fatty acid synthesis (O’Neill et al., 2013). Interestingly, the activity of *ACC* (more than 1,000 U/mg prot.) in liver of turtle was higher than that of *FAS* (about 10 U/mg prot.) in our study, suggesting that *ACC* is more stress-responsive in hepatic lipogenesis and subject to inhibition (or degradation) in *T. s. elegans* in response to salinity stress. Cytosolic *ACC1* is the rate-limiting enzyme in fatty acid synthesis, whereas *ACC2* co-localizes with mitochondria and its product, and malonyl-CoA serves to inhibit carnitine palmitoyl transferase (*CPT1*), an outer mitochondrial membrane protein that catalyzes fatty acid transport into mitochondria for oxidation (Hardie and Pan, 2002). Therefore, the increased expression of *CPT1* during increased salinity was in accordance with the reduced level of *ACC*.

*PPARα* is a major regulator of genes involved in fatty acid oxidation in liver mitochondria and peroxisomes (Kohjima et al., 2007; Monsalve et al., 2013). It regulates lipid oxidation enzymes in liver, such as *CPT-1*, *CPT-2*, *ACO*, *ACSL-1*, and liver fatty acid-binding protein (*L-FABP*; Kersten et al., 2000). The activated *AMPK* likely facilitated the observed increase of *PPARα* transcript level with increased salinity stress (Joly et al., 2010). Furthermore, the downstream lipid oxidation genes under *PPARα* regulation, *CPT-1*, *CPT-2*, *ACO*, and *ACSL-1*, were also significantly upregulated in liver of salinity-stressed turtles. Accordingly, the CPT activity in liver increased as salinity increased, whereas ACO activity peaked at S5 group, suggesting that the lower salinity stress was able to stimulate lipid oxidation, and ACO activity is not as involved in providing energy at higher salinity.

With exposed time extended, the expression of *ChREBP* was highest in S5 group at 48 h, and *SREBP-1c* was highest in S5 group at 24 h, while there was no significance between other groups. As for *PPARα*, the expression level was highest in S5 group at 48 h, and the same trend existed in S15 group. Also, there was no significance in the gene expressions of *SCD-1* and *CPT-2* between different exposed times, which indicated that *T. s. elegans* could adapt itself into salinity stress to some extend after longer exposure. However, some gene expressions and enzyme activities such as *CPT-1* decreased with exposure time extended.

### Content of Triglyceride, Low-Density Lipoprotein Cholesterol, High-Density Lipoprotein Cholesterol, and Total Cholesterol in Plasma During Salinity Stress

The activation of compensatory acclimation mechanisms *via* a substantial energetic reorganization over a relatively short period of time has been reported in other organisms such as euryhaline fish that experience osmotic stress (Kültz, 2015). Herein, several blood parameters were evaluated to analyze the potential effects of hyperosmotic stress on lipid-related products in blood. The levels of TG in plasma increased as salinity increased, which is consistent with increased levels plasma TG found in sea bream (*Sparus auratus*) after long-term seawater acclimation (AasHansen et al., 2005). Increase in TG level suggests an enhanced capacity for oxidizing lipids in the species. However, it should be noted that studies have found that plasma TG levels in sea bream and Arctic char (*S. alpinus*) did not change during 96-h acclimation to seawater, suggesting that lipid metabolism was unchanged (Nordgarden et al., 2002; Susana et al., 2005; Bystriansky et al., 2007). These inconsistencies between species may be partially due to acclimation salinities, exposure durations, and other experimental conditions.

By contrast, the plasma levels of LDL-C, HDL-C, and TC in S5 group did not change significantly, relative to control, which suggested that *T. s. elegans* could adapt to salinity under 5‰ while maintaining crucial blood parameters at homeostatic levels. However, the content of LDL-C and TC in S15 group increased significantly after 30 days of chronic, long-term salinity stress, potentially indicating that the energy balance of the organism was being affected. Furthermore, excessive LDL-C and TC in plasma has been shown to lead to endoplasmic reticulum stress (Jiang et al., 2015), and so when the environmental salinity increased up to 15‰, the salinity adaptation of *T. s. elegans* was challenged.

## Conclusion

**Figure 7:**
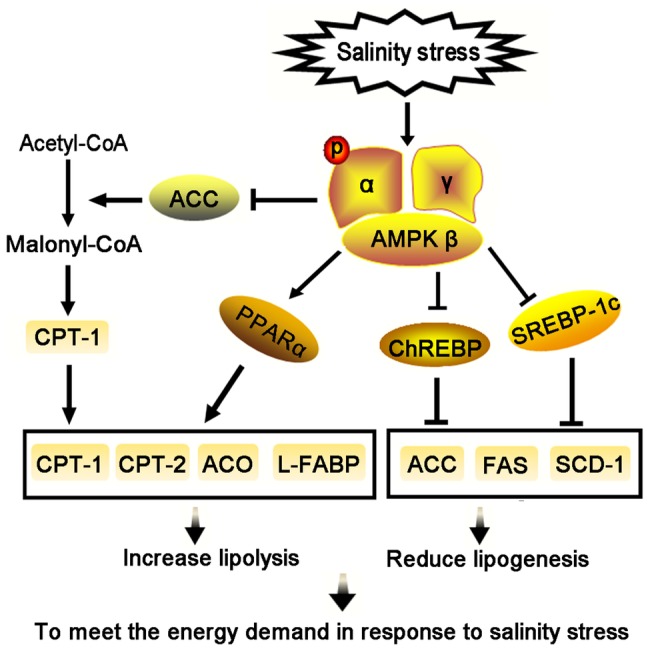
Salinity stress activates the AMPK signaling to regulate lipid metabolism in liver of *T. s. elegans*.

In our study, AMPK activity increased dramatically when *T. s. elegans* was subjected to increased ambient salinity, along with *AMPKα* mRNA levels. Salinity-activated AMPK signaling was shown to potentially inhibit the expression of *SREBP-1c* and *ChREBP* transcription factors, which led to a reduction in mRNA transcripts and enzymatic activities of their downstream targets, *ACC* and *FAS*. However, *AMPK* activation was found to promote the expression of *PPARα* that could, in turn, mediate upregulation of the expression of its target genes *CPT* and *ACO*. The *AMPK* signaling networks examined herein indicated an inhibition of lipogenesis and a promotion of lipid oxidation to meet the energy demand in response to salinity stress (Figure 7). The AMPK-mediated regulation of lipid metabolism in response to salinity sets the stage for future work on other molecular mechanisms involved in salinity tolerance and survival in *T. s. elegans.*

## Data Availability

The raw data supporting the conclusions of this manuscript will be made available by the authors, without undue reservation, to any qualified researcher.

## Ethics Statement

All experimental procedures had the prior approval of the Hainan Provincial Ecological Environment Education Center Experimental Animal Ethics Committee and were conducted under standard protocols for the care and use of laboratory animals at Hainan Normal University (No. HNECEE-2014-004).

## Author Contributions

All authors contributed to the preparation of this manuscript. MH conceived and designed the experiments, wrote the paper, and reviewed drafts of the paper. NL, JL, WL, and LL were integral in the collection and initial processing of turtle samples used for this study. NL, QL, and RW all contributed to the data collection and analysis in this study. KS and HS reviewed drafts of the paper and approved the final draft. LD contributed reagents, materials and analysis tools, prepared figures and tables, reviewed drafts of the paper, and approved the final draft. All authors have approved this statement and the final article.

### Conflict of Interest Statement

The authors declare that the research was conducted in the absence of any commercial or financial relationships that could be construed as a potential conflict of interest.
